# Three types of end-to-side microvascular anastomosis training models using rat common iliac arteries

**DOI:** 10.3389/fsurg.2023.1122551

**Published:** 2023-03-16

**Authors:** Zongyu Xiao, Ji Wang, Jingpeng Guo, Qi Pan

**Affiliations:** ^1^ Department of Neurosurgery, Dushu Lake Hospital Affiliated to Soochow University, Suzhou, China; ^2^ Department of Neurosurgery, The Second Affiliated Hospital of Guangzhou Medical University, Guangzhou, China; ^3^ Department of Neurosurgery, Fuyang People’s Hospital, Fuyang, China; ^4^ Department of Neurosurgery, The First Affiliated Hospital of Hainan Medical University, Haikou, China

**Keywords:** end-to-side, microanastomosis, training, rat, common iliac artery

## Abstract

**Background:**

: Instead of only practicing these perfectly matched end-to-side anastomoses in microsurgical laboratories, we must learn how to perform these so-called “imperfect” end-to-side anastomoses in the laboratory.

**Methods:**

Three types of end-to-side microvascular anastomoses using the rat common iliac artery (CIA), one with the proximal end of the CIA to the contralateral side of the CIA, another with the distal end of the CIA to the contralateral side of the CIA, and the third with the distal end of the CIA to the ipsilateral side of the common iliac vein (CIV), were presented to simulate different end-to-side anastomosis situations in a microsurgical laboratory. Diameters of CIA and CIV, distances between temporary clips, the length of arteriotomy or venotomy, and the distribution of stitches were recorded. The patency rates were evaluated immediately after the anastomosis was completed and 30 min later. After animal euthanasia, the donor vessel was cut close to the anastomotic site, and the orifice size and intimal attachment were evaluated by inspecting them through inside the vessel.

**Results:**

The diameters of the CIA and CIV were 0.8–1.2 mm and 1.2–1.5 mm, respectively. The end-to-side microvascular anastomosis arteriotomy or venotomy is approximately 2.00–2.50 mm, the distance between the aneurysm clips on the recipient CIA or CIV is approximately 4.00–7.00 mm, and the distance between the corner of the arteriotomy or venotomy and the temporary aneurysm clip was 1.00–3.00 mm. Three types of end-to-side anastomoses using the CIA were successfully performed, and 100% patency rates were achieved immediately and 30 min postoperatively. Good distribution of stitches, wide orifice, and intimal attachment were recorded in the study in all groups.

**Conclusions:**

Three types of end-to-side anastomoses using rat CIAs could be efficiently used to mimic three different anastomotic situations.

## Introduction

**Figure 1:**
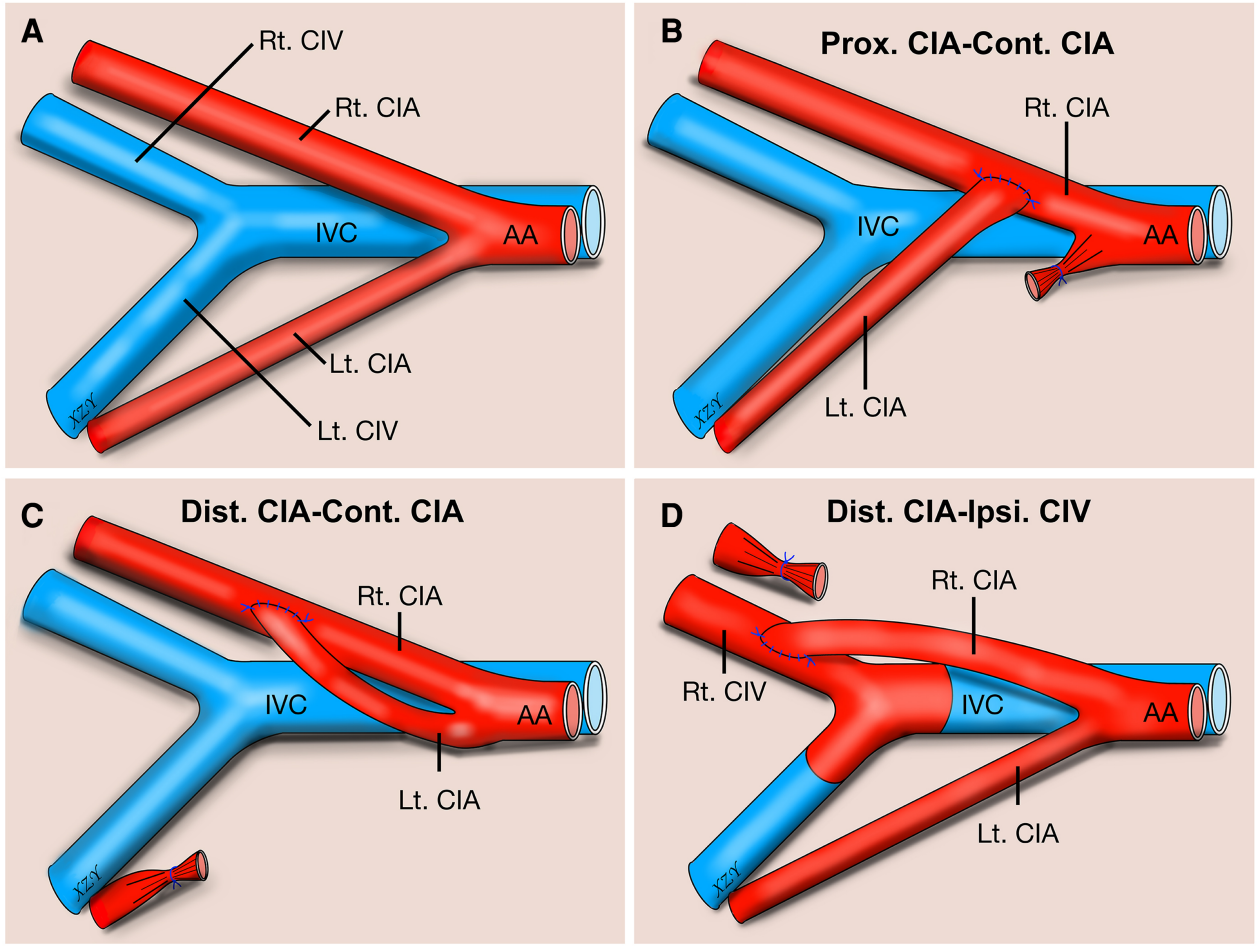
Schematic illustration about three types of end-to-side microvascular anastomosis training model using rat common iliac artery. Anatomy schematic illustration of the rat CIA regarding three types of end-to-side microvascular anastomosis training model using rat CIA (**A**). Prox. CIA to contralateral CIA end-to-side anastomosis (**B**). Distal CIA to contralateral CIA end-to-side anastomosis (**C**). Distal CIA to ipsilateral CIV end-to-side anastomosis (**D**). AA, abdominal aorta; CIA, common iliac artery; CIV, common iliac vein; Cont., contralateral; Dist., distal; Ipsi., Ipsilateral; IVC, inferior vena cava; Lt., left; Prox., proximal; Rt., right.

End-to-side microvascular anastomosis is one of the most commonly used anastomotic configurations in cerebral revascularization surgeries and connects the end of the donor artery with the side of the recipient artery through an anastomosis (1–5). Theoretically, the ideal anastomosis should be performed between two perfectly matched vessels under little or no tension situations; however, nonideal anastomosis, such as mismatched vessels or some tension between donor and recipient vessels, is commonly encountered in real operations (6, 7). Yarsagil emphasized that the microvascular anastomosis technique should be fully mastered in microsurgical training laboratories before its application in human beings (7, 8). Therefore, instead of only practicing these perfectly matched end-to-side anastomoses in microsurgical laboratories, we must learn how to perform these so-called “imperfect” end-to-side anastomoses in the laboratory. Previously, many types of end-to-side microvascular anastomosis training models have been described, such as those that are ipsilateral to contralateral common carotid arteries (9, 10), common carotid artery to external jugular vein (11, 12), ipsilateral to contralateral common iliac arteries (7, 9), and femoral artery to vein (13–15). In this article, three types of end-to-side microvascular anastomotic configurations using the rat common iliac artery (CIA), one with the proximal end of the CIA to the contralateral side of the CIA (Prox. CIA-Cont. CIA), another with the distal end of the CIA to the contralateral side of the CIA (Dist. CIA-Cont. CIA), and the third with the distal end of the CIA to the ipsilateral side of the common iliac vein (CIV) (Dist. CIA-Ipsi. CIV), were presented to simulate different end-to-side anastomosis situations in a microsurgical laboratory (Figure 1).

## Methods and materials

Forty-eight male Sprague-Dawley (S-D) rats weighing 200–250 g (Beijing Vital River Laboratory Animal Technology Company, Beijing, China) were used in the study. Blue, nonabsorbable monofilament polypropylene 10-0 sutures (W2790, 13 cm in length, 3.8 mm, 3/8 circle taper point, BV 75-3, Ethicon) were used to perform microvascular anastomosis under a training microsurgical microscope (Zeiss, OPMI Pico; Carl Zeiss Meditec AG, Jena, Germany). Microsurgical instruments were purchased from Shanghai Medical Instruments Co., Ltd., Shanghai, China, including two microforceps with 0.15 mm (WA3060) and 0.3 mm (WA3070) tips, straight or curved microscissors (WA1050 and WA1060), and straight or curved microneedle holders (WA2050 and WA2060). All procedures were performed by the first author (ZX) using 10× or 16× magnification *via* a microscope**.** In the study, the rats were anesthetized intraperitoneally with pentobarbital (50 mg/kg), and they were euthanized by an intraperitoneal injection of pentobarbital (200 mg/kg) at the end of the experiment. No systemic anticoagulant agents or antibiotics were used in the study.

### Ethical statement

The study was carried out in compliance with the ARRIVE (Animal Research: Reporting of *In Vivo* Experiments) guidelines, and all animal experiments were performed according to the national and international guidelines and were approved by the Research Ethics Committee.

### Proximal CIA to contralateral CIA end-to-side anastomosis

**Figure 2:**
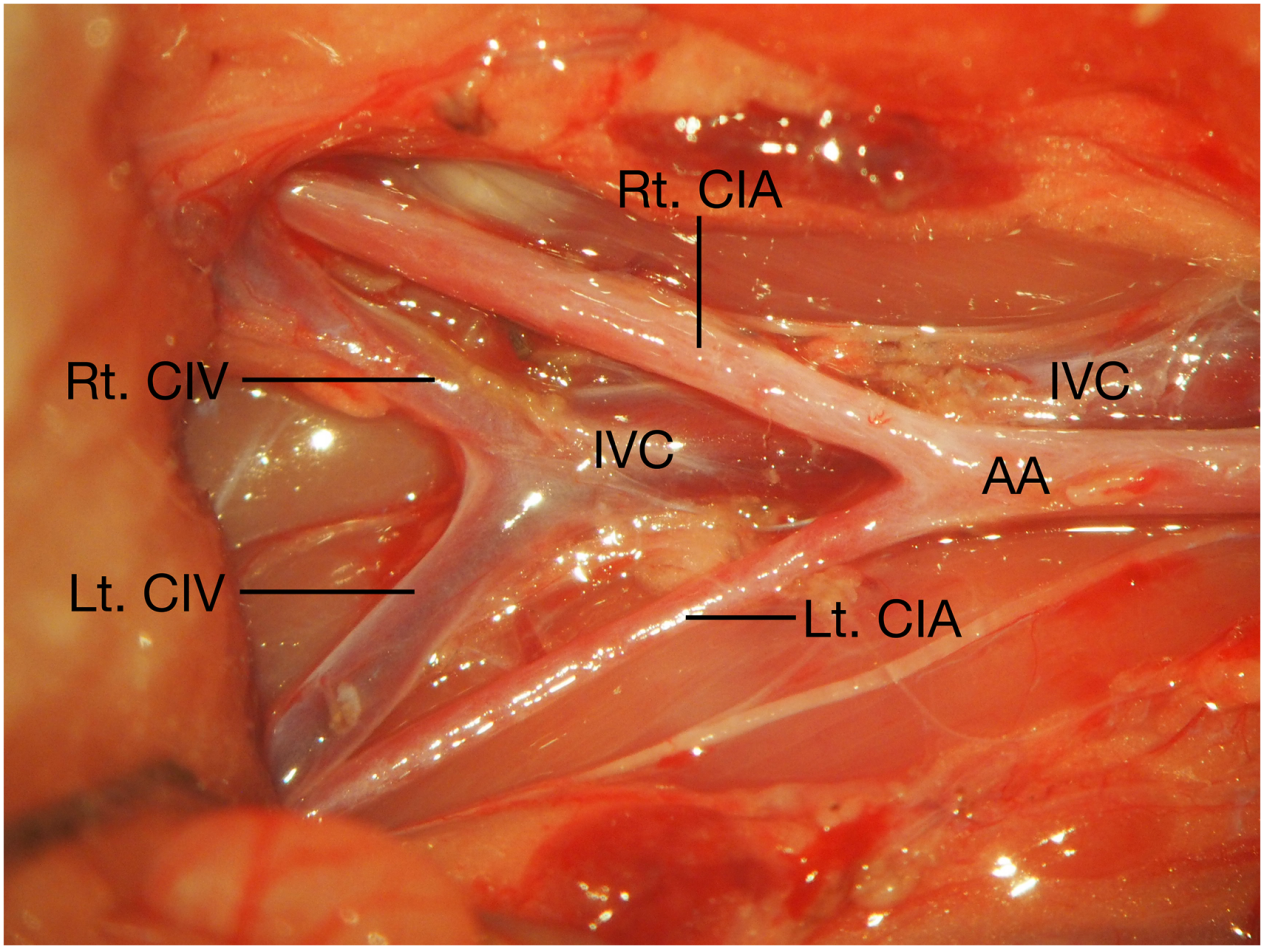
Anatomy of the CIA, CIV, IVC, and AA related to the end-to-side microvascular anastomosis using rat CIA. AA, abdominal aorta; CIA, common iliac artery; CIV, common iliac vein; IVC, inferior vena cava; Lt., left; Rt., right.

**Figure 3:**
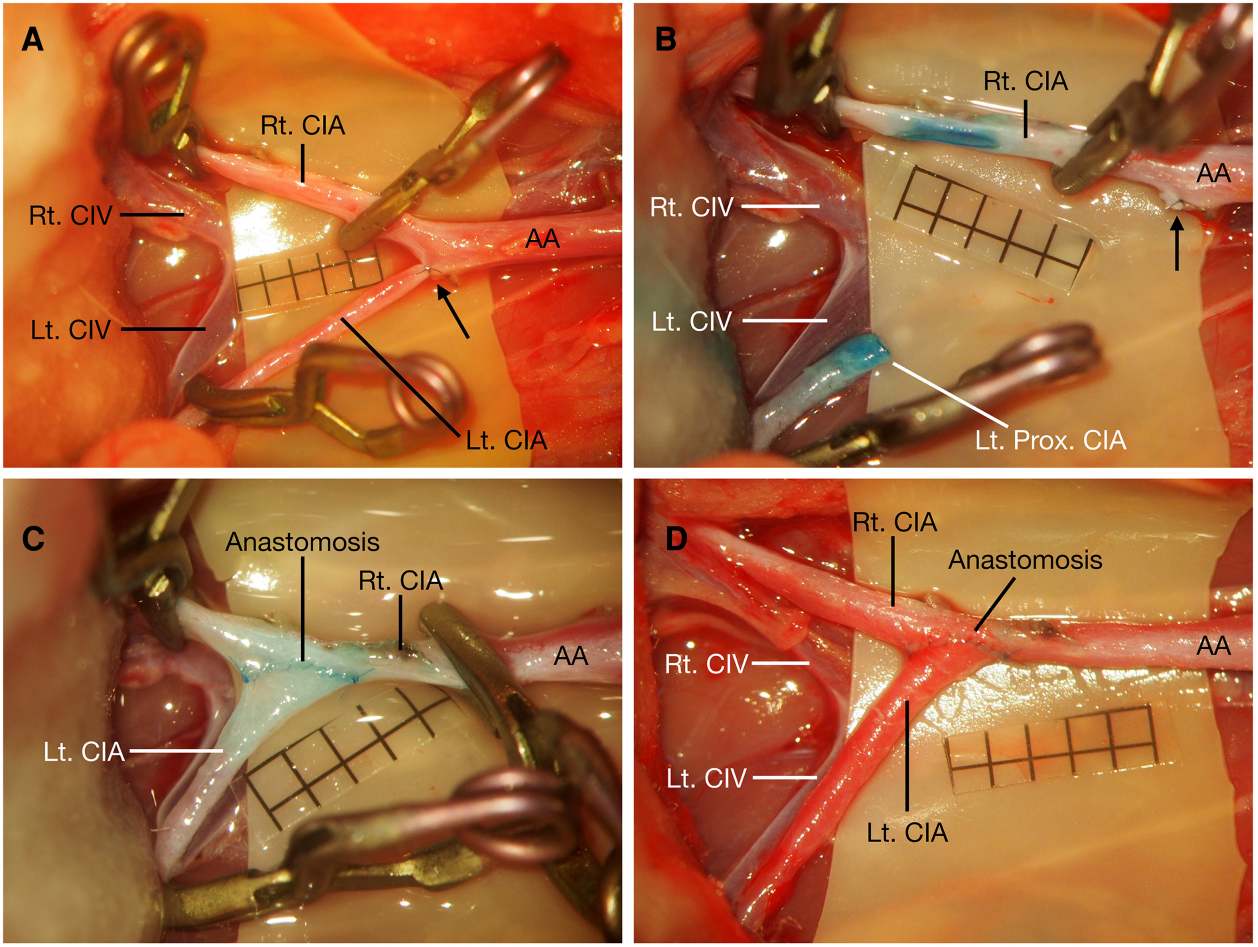
Proximal CIA to contralateral CIA end-to-side anastomosis. After bilateral CIAs were carefully dissected, the right CIA was temporarily occluded, the distal part of the left CIA was temporarily occluded, and the proximal part was ligated at its origin (black arrow) (**A**). The proximal left CIA was divided distal to the ligation point (black arrow), and the cutting end of the CIA was cut in a fish-mouth fashion; an arteriotomy was made on the right CIA (**B**). Proximal CIA to contralateral CIA end-to-side anastomosis was performed (**C**), and blood flow was restored by releasing these temporary clips (**D**). Scale bar = 1 mm. AA, abdominal aorta; CIA, common iliac artery; CIV, common iliac vein; Lt., left; Prox., proximal; Rt., right.

The animals were placed supine after anesthetization, and a midline abdominal skin incision was made. The bilateral CIAs were carefully and fully dissected from their abdominal aorta (AA) origin to their distal bifurcation into the internal and external iliac arteries, and the connective tissue around the CIA was meticulously dissected until the CIA was completely freed from them and the underling CIVs. Any tiny branches from the CIA were meticulously coagulated with fine-tipped bipolar forceps and were divided with microscissors (Figures 1A, 2). After the bilateral CIAs were fully isolated, the distal part of the left CIA was temporarily occluded with an aneurysm clip, the proximal part was ligated using 10-0 nylon sutures at its origin from the AA, and then the left CIA was transected just distal to the ligation point. The right CIA was temporarily occluded with two aneurysm clips (Figure 3A). The proximal cutting end of the left CIA was trimmed in an oblique fashion, and a linear arteriotomy with the same length as the left proximal CIA oblique cutting end was made on the anterior surface of the right CIA (Figure 3B). The adventitia around the cutting end of the left CIA and the arteriotomy on the right CIA were carefully removed. An end-to-side anastomosis was performed as we previously described (7). Two staying sutures were placed at the corner of the arteriotomy to draw the donor and recipient arteries together. Then, the posterior wall of the anastomosis was closed in an intraluminal continuous suturing technique. After that, the anterior wall was closed in an extraluminal continuous suture pattern, the depth of the sutures was one to two times the thickness of the CIA wall, and the spacing was approximately 3–5 sutures per millimeter. After completion of the anastomosis (Figure 3C), blood flow was restored by removing the temporary clips (Figures 1B, 3D). The patency of the anastomosis was evaluated using Acland's test (6, 7, 16–18), which was carried out by milking the distal part of the left CIA with microforceps to empty and refill it with bypass flow (Supplementary Video 1).

### Distal CIA to contralateral CIA end-to-side anastomosis

**Figure 4:**
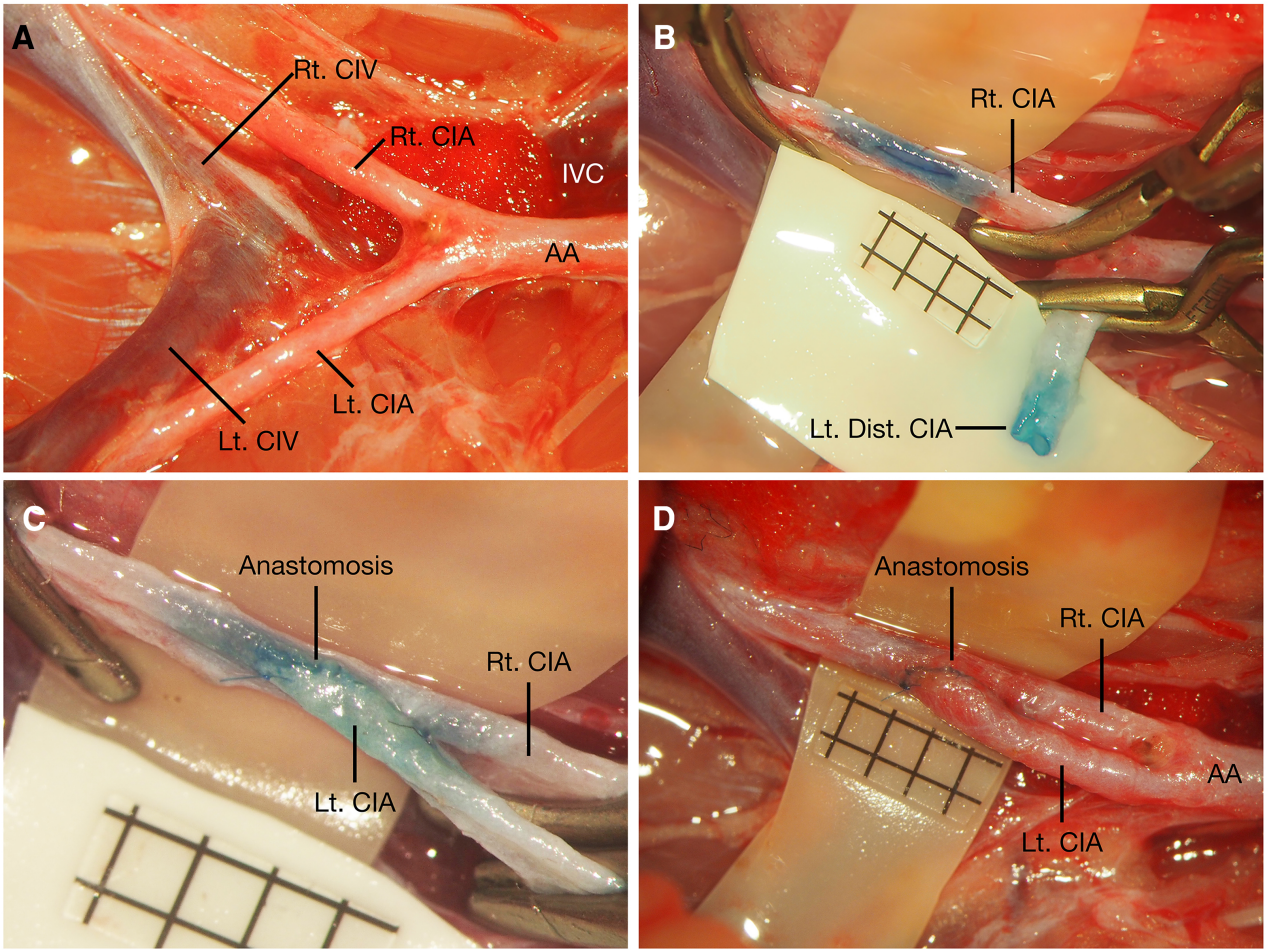
Distal CIA to contralateral CIA end-to-side anastomosis. After bilateral CIAs were carefully dissected (**A**), the right CIA was temporarily occluded, the proximal part of the left CIA was temporarily occluded, and the distal part was ligated before its distal bifurcation; the cutting end of the left CIA was cut in fish-mouth fashion, and an arteriotomy was made on the right CIA (**B**). Distal CIA to contralateral CIA end-to-side anastomosis was performed (**C**), and blood flow was restored by releasing these temporary clips (**D**). Scale bar = 1 mm. AA, abdominal aorta; CIA, common iliac artery; CIV, common iliac vein; Dist., distal; IVC, inferior vena cava; Lt., left; Rt., right.

After the bilateral CIAs were fully dissected from their origin to their distal bifurcations (Figure 4A), the proximal part of the left CIA was temporarily clipped at its origin from the AA with an aneurysm clip, the distal part of the left CIA was ligated using 10-0 nylon sutures just before the bifurcation to the internal and external iliac arteries, and then it was transected proximal to the ligation point. The cutting end of the left CIA was trimmed in a fish-mouth fashion. The right CIA was temporarily occluded with two aneurysm clips, and a linear arteriotomy with the same length as the left distal CIA fish-mouthed cutting end was made on the right CIA (Figure 4B). After the completion of two staying sutures at the corner of the arteriotomy, an end-to-side anastomosis was performed using a traditional extraluminal interrupted or continuous suturing technique. The depth of the sutures and the spacing were the same as those in the Prox. CIA-Cont. CIA end-to-side anastomosis. After the anterior wall was closed, the posterior wall could be easily exposed by rotating the left CIA to the other side without obvious tension. Then, the posterior wall was placed by facing the surgeon and was sutured with the same extraluminal suturing technique (Figure 4C). After the blood flow of the anastomosis was restored (Figures 1C, 4D), the patency of the anastomosis was evaluated using Acland's test (6, 7, 16–18) on the distal part of the right CIA, while the right CIA proximal to the anastomosis was temporarily occluded (Supplementary Video 2).

### Distal CIA to ipsilateral CIV end-to-side anastomosis

**Figure 5:**
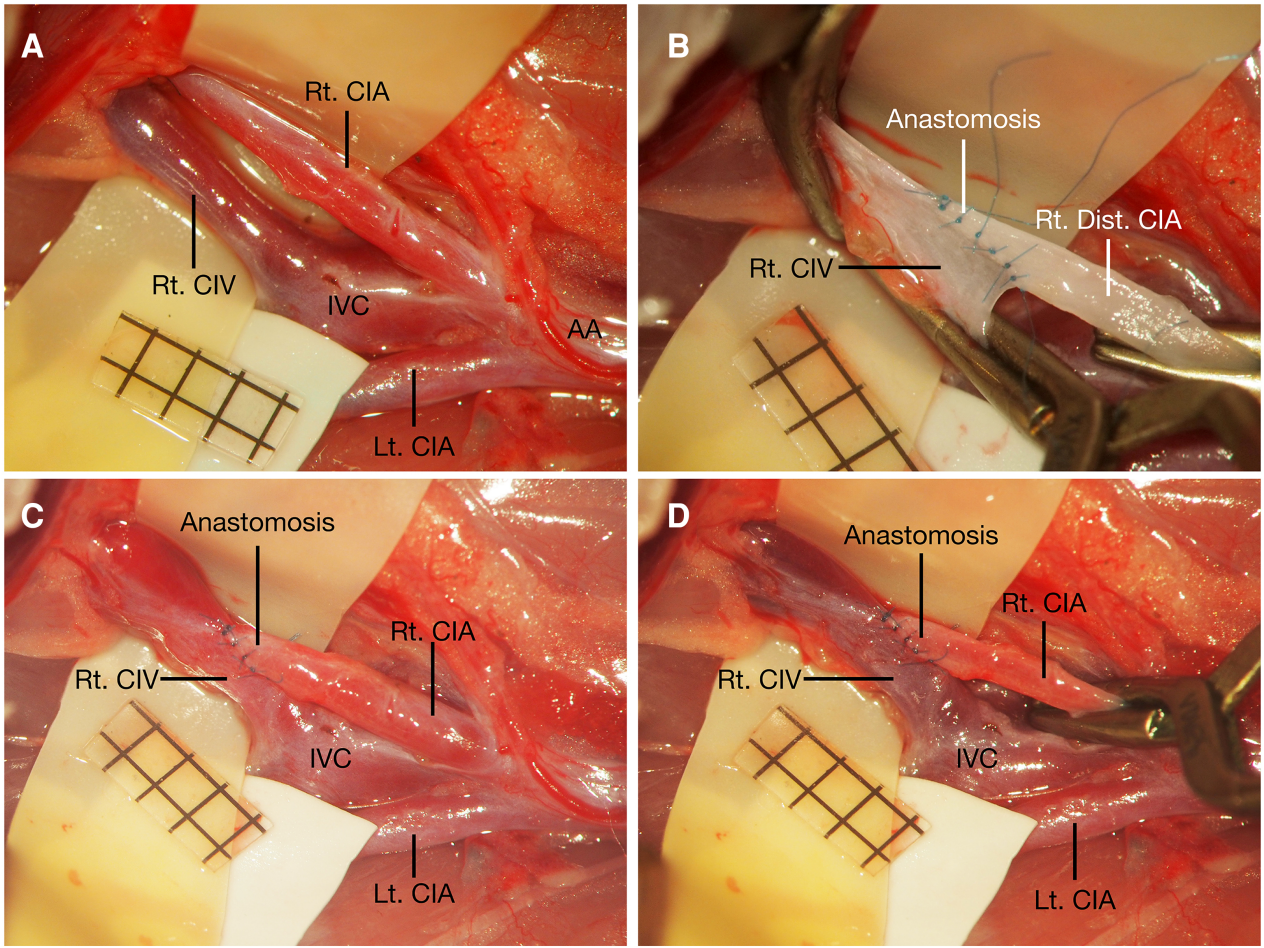
Distal CIA to ipsilateral CIV end-to-side anastomosis. After right CIA and CIV were fully dissected (**A**), the distal cutting end of the right CIA was sutured to the side of the right CIV (**B**). After restoration of blood flow, right CIV appeared to be bright red-colored and expanded through patent anastomosis due to arterial blood flow from CIA (**C**), and the color of the right CIV turned back to dark red again when the right CIA was temporarily occluded (**D**). Scale bar = 1 mm. AA, abdominal aorta; CIA, common iliac artery; CIV, common iliac vein; IVC, inferior vena cava; Lt., left; Rt., right.

The right CIA and CIV were fully dissected from their origin to their distal bifurcations, and any branches arising from the segment were meticulously coagulated and divided (Figure 5A). The proximal part of the right CIA was temporarily clipped, and the distal part of the right CIA was ligated using 10-0 nylon sutures just before the bifurcation into the internal and external iliac arteries. Then, the CIA was transected proximal to the ligation point, and the cutting end of the right distal CIA was cut in a fish-mouth fashion. Then, the right CIV was temporarily occluded with two aneurysm clips, and a linear venotomy with the same length as the right CIA fish-mouthed cutting end was made on the right CIV. Then, the distal end of the right CIA was anastomosed to the side of the CIV (Figure 5B). There was no obvious tension in the Dis. CIA-Ipsi. CIV, the right CIA could be rotated easily, and the anastomosis was performed using a traditional extraluminal interrupted or continuous suturing technique. The depth of the sutures was one to two times the thickness of the CIA wall, and the spacing was approximately 3–5 sutures per millimeter. After blood flow of the anastomosis was restored (Figures 1D, 5C), the patency of the anastomosis was evaluated using Acland's test (6, 7, 16–18) on the distal portion of the CIV (Supplementary Video 3).

### Patency evaluation

The patency of the anastomosis was evaluated through direct observation under a microscope or Acland's test immediately after the anastomosis was completed and 30 min later.

### Measurement

**Figure 6:**
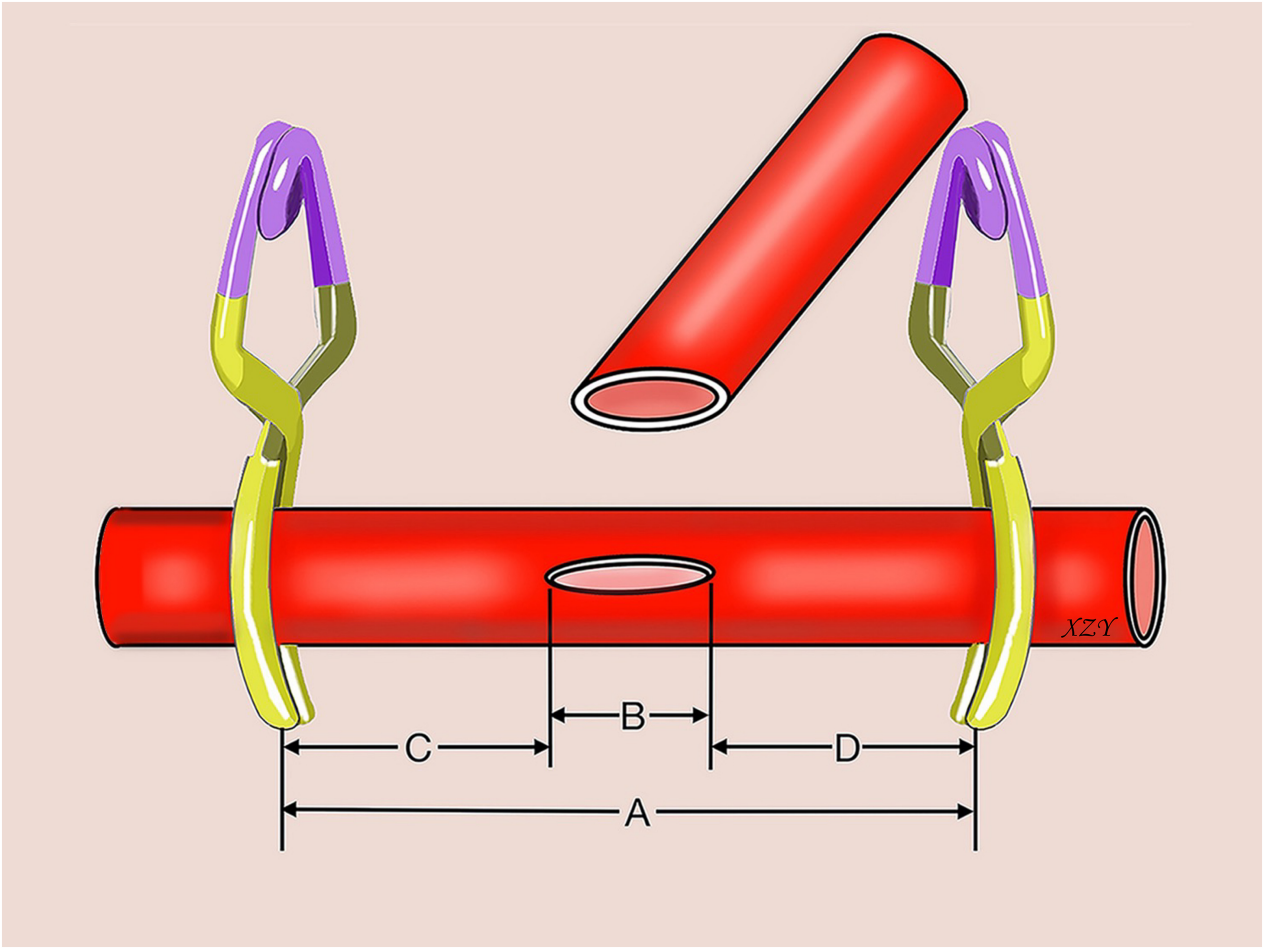
Schematic illustration shows the measurements in end-to-side anastomosis using rat common iliac arteries. (**A**) distance between two aneurysm clips; (**B**) length of the arteriotomy or venotomy; (**C**) distance between the proximal aneurysm clip and the proximal end of the arteriotomy or venotomy; (**D**) distance between the distal aneurysm clip and the distal end of the arteriotomy or venotomy.

The diameters of the CIA and CIV were recorded. The total suturing time, the distribution of stitches, the distance between the two temporary aneurysm clips on the recipient vessel, the length of the arteriotomy or venotomy, and the distance between the corner of the arteriotomy or venotomy and the temporary clips were recorded (Table 1, Figure 6). After animal euthanasia, the donor vessel was cut close to the anastomotic site, and the orifice size and intimal attachment were evaluated by inspecting them through inside the vessel, as suggested by Hafez et al. (19).

**Table 1:** Measurements in end-to-side microvascular anastomosis using rat CIA.

Measurement (mm)	Prox. CIA-Cont. CIA 1 (*n* = 12)		Dist. CIA-Cont. CIA 2 (*n* = 12)		Dist. CIA-Ipsi. CIV (*n* = 12)	
	Mean ± SD	Range	Mean ± SD	Range	Mean ± SD	Range
(A) Distance between two aneurysm clips on the recipient vessel	5.59 ± 0.66	5.00–7.00	5.72 ± 0.57	5.00–7.00	5.40 ± 0.78	4.00–6.20
(B) Length of arteriotomy or venotomy	2.16 ± 0.22	2.00–2.50	2.18 ± 0.21	2.00–2.50	2.09 ± 0.12	2.00–2.30
(C) Distance between proximal aneurysm clip and proximal end of arteriotomy or venotomy	1.71 ± 0.42	1.00–2.50	1.75 ± 0.26	1.50–2.00	1.48 ± 0.44	1.00–2.00
(D) Distance between distal aneurysm clip and distal end of arteriotomy or venotomy	1.73 ± 0.64	1.00–3.00	1.79 ± 0.45	1.00–2.50	1.83 ± 0.44	1.00–2.50
(E) Diameter of left CIA	0.94 ± 0.09	0.80–1.00	0.98 ± 0.10	0.80–1.20		
(F) Diameter of right CIA	1.13 ± 0.08	1.00–1.20	1.13 ± 0.08	1.00–1.20	1.05 ± 0.09	1.00–1.20
(G) Diameter of right CIV					1.33 ± 0.10	1.20–1.50

SD, standard deviation; CIA, common iliac artery; CIV, common iliac vein; Cont., contralateral; Dist., Distal; Ipsi., ipsilateral; Prox., Proximal.

### Statistical analysis

The data are presented as the means centerstandard deviation (SD). The analysis was carried out using SPSS 19.0 (SPSS, Inc., Chicago, IL, United States).

## Results

**Figure 7:**
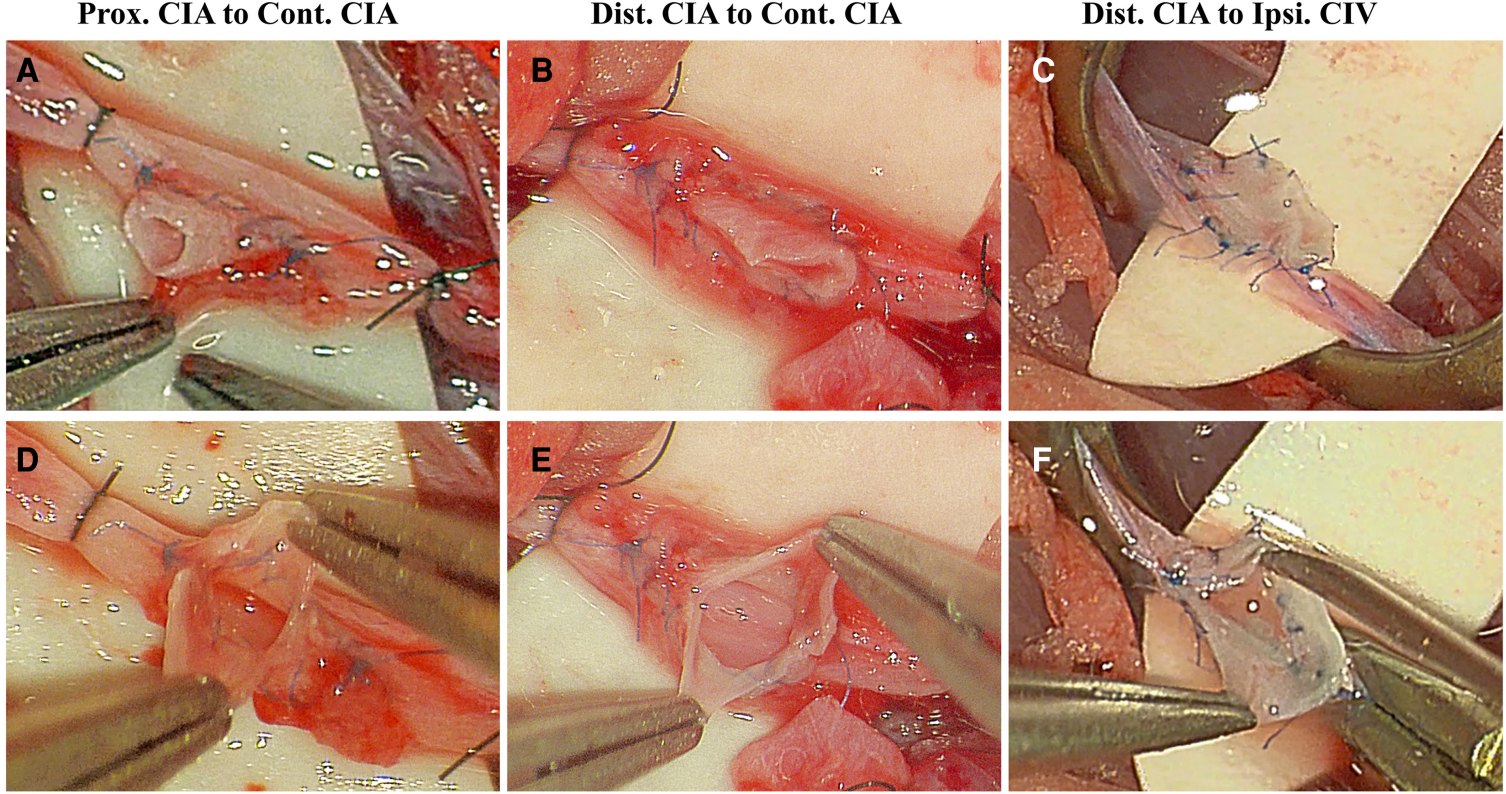
Distribution of stitches, orifice size, and intimal attachment were inspected in end-to-side anastomosis using rat common iliac arteries. Good distribution of stitches, wide orifice and intimal attachment were recorded in Prox. CIA-Cont. CIA (**A,C**), Dist. CIA-Cont. CIA (**B,D**), and Dist. CIA-Ipsi. CIV (**C,F**) end-to-side anastomosis. CIA, common iliac artery; CIV, common iliac vein; Cont., contralateral; Dist., distal; Ipsi., Ipsilateral; Prox., proximal.

In this study, the diameters of the CIA and CIV were 0.8–1.2 mm and 1.2–1.5 mm, respectively (Table 1). No animal seizures or animal deaths were recorded during the procedure. Three types of end-to-side anastomosis using the CIA were successfully performed. No thrombus or anastomotic aneurysms were observed at the end-to-side anastomosis sites, and 100% patency rates were achieved immediately and 30 min postoperatively. Pulsation of the artery or vein distal to the anastomosis was observed under a microscope after blood flow was restored in all groups. In the Dis. CIA-Ipsi. CIV end-to-side anastomosis, the CIV appeared to be bright red-colored and expanded through the patent anastomosis due to the arterial blood flow from the CIA, and the color of the CIV turned back to dark red again when the CIA was temporarily occluded (Figure 5D). Good distribution of stitches, wide orifice, and intimal attachment were recorded in the study in all groups. The thread was directly exposed in some of the anastomoses, especially in the Prox. CIA-Cont. CIA anastomosis when intraluminal suturing technique was used to close the posterior wall (Figure 7).

In the study, 12 cases of Prox. CIA-Cont. CIA anastomosis (*n* = 12), 12 cases of Dist. CIA-Cont. CIA anastomosis (*n* = 12), and 12 cases of Dist. CIA-Ipsi. CIV anastomosis (*n* = 12) were performed with a continuous suturing technique, and 6 cases of Dist. CIA-Cont. CIA anastomosis and 6 cases of Dist. CIA-Ipsi. CIV anastomosis were performed using the interrupted suturing technique. The total suturing time for Prox. CIA-Cont. CIA, Dist. CIA-Cont. CIA, and Dist. CIA-Ipsi. CIV anastomosis using the continuous suturing technique was approximately 27.75 ± 1.36 min (26–30 min, *n* = 12), 25.33 ± 2.19 min (21–28 min, *n* = 12), and 27.58 ± 2.07 min (25–31 min, *n* = 12), respectively. The total suturing time for Dist. CIA-Cont. CIA and Dist. CIA-Ipsi. CIV anastomosis using the interrupted suturing technique was approximately 32.00 ± 2.97 min (27–35 min, *n* = 6) and 32.50 ± 3.39 min (28–36 min, *n* = 6), respectively.

## Discussion

End-to-side microvascular anastomoses are one of the most commonly used anastomotic configurations in cerebral revascularization surgeries, especially extracranial to intracranial bypass surgeries, and some of these anastomoses include anastomoses of the superficial temporal artery (STA)-middle cerebral artery (MCA) (1), STA-anterior cerebral artery (ACA) (2), STA-superior cerebellar artery (SCA) or STA-posterior cerebral artery (PCA) (3), occipital artery (OA)-posterior inferior cerebellar artery (PICA) (4), OA-anterior inferior cerebellar artery (AICA) (5), etc. Theoretically, the ideal end-to-side microvascular anastomosis should be performed between two perfectly matched arteries under tension-free suturing conditions; however, different nonideal anastomotic situations are encountered in real operations and include different degrees of tension and discrepancies in the diameter, thickness, texture and consistency between the donor and recipient arteries, vessel direction, surgical corridor and depth, etc. (6, 7, 16, 18). Yarsagil emphasized that the microvascular anastomosis technique should be fully mastered in microsurgical laboratories before its application in human beings (7, 8). Therefore, instead of performing end-to-side microvascular anastomosis between two perfectly matched vessels, we also need to learn how to perform these so-called “imperfect” end-to-side anastomoses between mismatched vessels or under vascular tension in a microsurgical laboratory. In this article, three types of end-to-side microvascular anastomosis using the rat common iliac artery were presented to simulate three different anastomotic situations.

There are many types of end-to-side microvascular anastomosis training models, such as those that are ipsilateral to contralateral common carotid arteries (9, 10), common carotid artery to external jugular vein (11, 12), ipsilateral to contralateral common iliac arteries (7, 9), and femoral artery to vein (13–15). Previously, Tayebi Meybodi et al. (9), Hall (20), and we (7) have described Prox. CIA-Cont. CIA microvascular anastomosis for end-to-side microvascular anastomosis training; however, Dist. CIA-Cont. CIA and Dist. CIA-Ipsi. CIV end-to-side anastomosis have not been reported for anastomosis training. Due to the anatomical features, the rat CIA and CIV are tightly adhered to each other due to the thick layer of connective tissue between them, which makes the CIA and CIV dissection more difficult than dissection of the rat common carotid artery (9). In addition, multiple small branches from the CIA and CIV need to be coagulated and divided to fully isolate them; thus, dissection of the CIA and CIV carries the risk of potential vascular injury and hemorrhage, which may not be suitable for beginners of microvascular training when compared with the rat common carotid artery and femoral artery (9). However, delicate meticulous microsurgical dissection techniques are a prerequisite in neurosurgical interventions, especially when dealing with vital fragile structures. Tayebi Meybodi et al. mentioned that dissection of the aortoiliac arteries and their accompanying veins was very similar to the dissection of the middle cerebral artery and the adjacent Sylvian veins in the Sylvian fissure (21). Therefore, the end-to-side microvascular anastomosis training model using rat CIA provides trainees with a similar Sylvian fissure dissection opportunity before performing a CIA end-to-side anastomosis; moreover, we believe that coagulation of these small branches of CIA and CIV also provides trainees with another chance to learn how to perform meticulous coagulation using bipolar coagulation under high magnification.

Generally, we should always try to perform a microvascular anastomosis without placing the vessels under tension or only with little tension (7). Full isolation of the donor and recipient vessels could maximize the exposure and minimize the tension of the anastomosis. In this study, to achieve a sufficient length of the donor CIA to minimize the tension between the donor CIA and recipient CIA or CIV during anastomosis, we fully dissected the donor CIA from its origin from the AA to its most distal portion before its bifurcation into the internal and external iliac arteries. After full isolation of the donor and recipient vessels, the microvascular anastomosis working space is also important. As we described previously in a side-to-side microvascular anastomosis training model using rat cervical vessels, the minimum distance between the corner of the arteriotomy or venotomy and the temporary aneurysm clip should be at least 1 mm to guarantee the following side-to-side microvascular anastomotic procedures (6). In the end-to-side microvascular anastomosis study, we found that the rule is also applicable; the end-to-side microvascular anastomosis arteriotomy or venotomy is approximately 2.00–2.50 mm; and the distance between the aneurysm clips on the recipient CIA or CIV is approximately 4.00–7.00 mm, which is slightly longer than the length of the arteriotomy or venotomy. The distance between the corner of the arteriotomy or venotomy and temporary aneurysm clip was 1.00–3.00 mm, but the limited distance had no obvious side effects on the required surgical space in the end-to-side microvascular anastomosis procedures.

Regardless of whether the CIA was transected either distally or proximally, the CIAs retracted significantly. Tayebi Meybodi et al. reported that the CIA retracts to almost one-third of its original length (9). Although the transected donor CIA retracted significantly, it had no obvious tension on the vessels or the anastomosis sites in the Dist. CIA-Cont. CIA and the Dist. CIA-Ipsi. CIV end-to-side anastomosis; we can easily put the distal cutting end of the donor CIA to the contralateral CIA or ipsilateral CIV under tension-free situations, and the donor CIA can be rotated easily. Thus, both sides of the anastomosis can be performed using either interrupted or continuous traditional extraluminal suturing techniques. Therefore, the Dist. CIA-Cont. CIA and Dist. CIA-Ipsi. CIV end-to-side anastomoses mimic the traditional extraluminal suturing end-to-side anastomosis between two perfectly matched arteries under tension-free situations. However, a lot of tension was encountered in drawing the proximal cutting end of the donor CIA to the contralateral CIA in the Prox. CIA-Cont. CIA end-to-side model; the rotation of the anastomosis may place the vessel at high risk of injury due to undue tension. It was impossible to suture the posterior wall of the anastomosis with an extraluminal suturing technique, and all of posterior wall in our Prox. CIA-Cont. CIA end-to-side microvascular anastomosis was performed through an intraluminal continuous suturing technique. The intraluminal suturing technique is also called the “*in situ* suturing technique,” and it is an effective suturing technique to close the posterior wall of the anastomosis when the vessels cannot be rotated (7). However, the intraluminal suturing technique is more challenging than the extraluminal suturing technique, and the risk of injury to the vascular endothelium is relatively high. Thus, its anastomotic thrombosis rate is higher than that of the traditional extraluminal suturing technique, but proficiency in the intraluminal suturing technique can be gained in microsurgical training laboratories (6, 7). Tayebi Meybodi et al. tried to rotate the vessel and tried to suture them extraluminally in the same CIA end-to-side anastomosis training model, and the same findings were reported. The posterior wall could not be completed extraluminally in any of their anastomosis training rats due to the high amount of tension on the CIA, and they also sutured the posterior wall in the intraluminal suturing technique (9). However, in 1980, Hall (20) performed the same CIA end-to-side anastomosis with the help of an Acland approximator. After the completion of the anterior wall in the extraluminal suturing technique, they successfully rotated the donor CIA to the other side and held it with the Acland approximator. Then, the other side of the anastomosis was sutured extraluminally. Hall (20) accepted that some tension was transmitted to the donor CIA distally by rotating the vessel using the Acland approximator, but the anastomosis site was tension-free. In this way, they achieved 100% patency rates immediately and 2 weeks after the procedure in 25 consecutive end-to-side anastomoses. Although Hall successfully performed the anastomosis under tension-free conditions in the anastomosis site in the extraluminal technique using an Acland approximator, the high tension on the vessel may cause it to have a high risk of injury. We suggest that the posterior wall of the Prox. CIA-Cont. CIA end-to-side anastomosis should be performed intraluminally if too much tension is encountered when rotating the vessel. Thus, the Prox. CIA-Cont. CIA end-to-side anastomosis model mimics the arterial end-to-side anastomosis between perfectly matched vessels under much tension, which requires an intraluminal suturing technique.

Theoretically, the diameter, thickness, texture, and consistency of the donor and recipient arteries should be matched perfectly (6). In the study, the Dist. CIA-Cont. CIA and Prox. CIA-Cont. CIA arterial end-to-side anastomosis models mimic the end-to-side anastomosis between perfectly matched arteries. However, mismatched vessels are inevitably encountered in cerebrovascular bypass surgery even if there is careful preoperative planning (6, 22), and the difficulty of anastomosis increases when very thin-walled vessels are encountered (23). The artery-to-vein microvascular training model is a good model to mimic this situation because veins are thinner, more fragile, easier to damage, and more prone to collapse than arteries. Additionally, veins become transparent after the blood is removed from them; hence, veins require more precise and gentler manipulation (6). In the study, the Dist. CIA to Ipsi. CIV arteriovenous anastomosis model was used to mimic the situation. The CIV was obviously thinner than the CIA, and it became transparent during anastomosis. The anastomotic situations were very similar to the situation when the STA was anastomosed to a very thin-walled M4 segment of the MCA in Moyamoya disease. Due to the fragility of the CIV, anastomosis should be performed under tension-free conditions; otherwise, the CIV is easily damaged. In this model, a sufficient length segment of the CIA could be mobilized easily, similar to the Dist. CIA-Cont. CIA end-to-side anastomosis. Thus, the traditional extraluminal suturing technique could be used in the model, and this model mimics the anastomosis between vessels with discrepancies in their thickness and while under tension-free suturing conditions.

Despite the three types of end-to-side anastomosis using the rat CIA as we described above, there are still other types of end-to-side configuration designs. For example, De Carolis and Sepulveda (24) reported an end-to-side training model using the CIA. They ligated and transected the distal part of the right CIA and then brought the distal part of the CIA proximally toward the aorta. A longitudinal incision was made on the aorta at 3 mm before its bifurcation, and an end-to-side anastomosis was performed using 10-0 sutures between the CIA and the aorta. In their model, De Carolis and Sepulveda mimicked the situation between two arteries with different diameters. Based on the different diameters, thicknesses, textures and consistencies of the vessels, and the different degrees of tension between them, there are still many other combinations of anastomotic designs. Furthermore, the real anastomotic situation also varies according to many factors, including the direction of the recipient and donor vessels, the surgical corridor and depth, and the tilt of the surgical field (16, 18), which may render a simple anastomotic procedure difficult to perform under some specific surgical situations. Therefore, we suggest that we should train ourselves on the different anastomotic configurations under different surgical situations in microsurgical laboratories as much as possible so that we can handle these difficult anastomotic situations competently and confidently in real operations.

Regardless of the anastomotic configuration, the quality of microvascular anastomosis plays a key role in maintaining patency in the long term. Trainees should always follow the basic principles of microvascular anastomosis (16, 18). An experimental practical scale proposed by Hafez et al. (19) is valuable for evaluating the quality of anastomosis, including the vessel suturing time, stitch distribution, intimal attachment, and orifice size. The trainee may cut the vessel open and evaluate the anastomotic quality inside the vessel when a nonliving animal model is used or after the completion of anastomosis after animal anesthetization, which is not possible in live surgery. Hafez et al. (19) suggested that we should try to suture the vessel with an intima-to-intima pattern while embedding the thread inside the vessel wall because thread exposure to the bloodstream may result in anastomotic failure due to thrombosis. Hafez et al. (19) reported that the rate of thread exposure inside the lumen was approximately 34% in their end-to-side microvascular anastomotic procedure using chicken vessels and wet tubes. In our study, good intimal attachment was observed, but the thread was directly exposed in some of our anastomoses as Hafez et al. (19) reported, especially in almost every case of Prox. CIA-Cont. CIA anastomosis when the intraluminal suturing technique was used to close the posterior wall, similar to side-to-side microvascular anastomosis (6, 7). However, it seems that thread exposure had no obvious side effect on the patency rate in our study, with a 100% patency rate being achieved in all groups. Many microsurgical laboratory studies and clinical investigations have demonstrated that performing these anastomoses using the intraluminal continuous suturing technique could allow patency to be maintained over the long term (2, 6, 7, 16, 18), but more research is needed to investigate the relationship between intraluminal thread exposure and anastomotic patency in the long term.

In the Prox. CIA-Cont. CIA end-to-side microvascular anastomosis model, we restored the blood flow of both lower extremities of the animals, which did not cause obvious ischemic problems in the lower extremities because of the patent anastomosis. However, in the Dist. CIA-Cont. CIA and Dist. CIA-Ipsi. CIV end-to-side anastomosis, the donor CIA was transected distally, which may cause ischemic problems to this side of the lower extremity if the anastomosis needs to be observed in the long term. However, in this study, the patency of the anastomosis was observed only 30 min after the procedure, and the ischemic problem did not cause any side effects with regard to our training.

## Conclusion

Three types of end-to-side anastomosis using rat CIAs could be efficiently used to mimic three different anastomotic situations. We suggested that the trainee should start performing end-to-side anastomosis surgeries using the Dist. CIA-Cont. CIA end-to-side anastomosis to mimic perfectly matched arterial anastomosis under tension-free conditions. Then, the trainee may further their training using the intraluminal suturing technique in the Prox. CIA-Cont. CIA end-to-side anastomosis to mimic perfectly matched arterial anastomosis under a lot of tension, or by performing the arteriovenous Dist. CIA-Ipsi. CIV end-to-side anastomosis to mimic anastomosis between mismatched vessels without tension.

## Data Availability

The original contributions presented in the study are included in the article/**Supplementary Material**, further inquiries can be directed to the corresponding author.
