# Supplementary material for: Three types of end-to-side microvascular anastomosis training models using rat common iliac arteries
Source: Front Surg. 2023 Mar 16;10:1122551. doi: 10.3389/fsurg.2023.1122551 (PMC10062452; doi:10.3389/fsurg.2023.1122551)
Supplement: Supplementary file 1 [file Table1.docx]

**Table 1 Measurements in end-to-side microvascular anastomosis using rat CIA**

| **Measurement (mm)** | **Prox. CIA -Cont. CIA 1 (n=12)** | |  | **Dist. CIA-Cont. CIA 2 (n=12)** | |  | **Dist. CIA- Ipsi. CIV (n=12)** | |
| --- | --- | --- | --- | --- | --- | --- | --- | --- |
|  | **Mean ± SD** | **Range** |  | **Mean ± SD** | **Range** |  | **Mean ± SD** | **Range** |
| A: distance between two aneurysm clips on the recipient vessel | 5.59±0.66 | 5.00-7.00 |  | 5.72±0.57 | 5.00-7.00 |  | 5.40±0.78 | 4.00-6.20 |
| B: length of arteriotomy or venotomy | 2.16±0.22 | 2.00-2.50 |  | 2.18±0.21 | 2.00-2.50 |  | 2.09±0.12 | 2.00-2.30 |
| C: distance between proximal aneurysm clip and proximal end of arteriotomy or venotomy | 1.71±0.42 | 1.00-2.50 |  | 1.75±0.26 | 1.50-2.00 |  | 1.48±0.44 | 1.00-2.00 |
| D: distance between distal aneurysm clip and distal end of arteriotomy or venotomy | 1.73±0.64 | 1.00-3.00 |  | 1.79±0.45 | 1.00-2.50 |  | 1.83±0.44 | 1.00-2.50 |
| E: diameter of left CIA | 0.94±0.09 | 0.80-1.00 |  | 0.98±0.10 | 0.80-1.20 |  |  |  |
| F: diameter of right CIA | 1.13±0.08 | 1.00-1.20 |  | 1.13±0.08 | 1.00-1.20 |  | 1.05±0.09 | 1.00-1.20 |
| G: diameter of right CIV |  |  |  |  |  |  | 1.33±0.10 | 1.20-1.50 |

CIA, common iliac artery; CIV, common iliac vein; Dist., Distal; Ipsi., ipsilateral; Prox., Proximal.
